# Conventional management has a greater negative impact on *Phaseolus vulgaris* L. rhizobia diversity and abundance than water scarcity

**DOI:** 10.3389/fpls.2024.1408125

**Published:** 2024-07-01

**Authors:** Arantza del-Canto, Alvaro Sanz-Saez, Katy D. Heath, Michael A. Grillo, Jónathan Heras, Maite Lacuesta

**Affiliations:** ^1^ Department of Plant Biology and Ecology, Pharmacy Faculty, University of the Basque Country, Universidad del País Vasco/Euskal Herriko Unibertsitatea (UPV/EHU), Vitoria-Gasteiz, Spain; ^2^ Department of Crop, Soil, and Environmental Sciences, Auburn University, Auburn, AL, United States; ^3^ Department of Plant Biology, University of Illinois, Urbana, IL, United States; ^4^ Department of Biology, Loyola University Chicago, Chicago, IL, United States; ^5^ Department of Mathematics and Computer Science, University of La Rioja, Logroño, Spain

**Keywords:** genomic fingerprinting, nodulation, organic management, common bean, strain diversity, yield

## Abstract

**Introduction:**

Drought is one of the biggest problems for crop production and also affects the survival and persistence of soil rhizobia, which limits the establishment of efficient symbiosis and endangers the productivity of legumes, the main source of plant protein worldwide.

**Aim:**

Since the biodiversity can be altered by several factors including abiotic stresses or cultural practices, the objective of this research was to evaluate the effect of water availability, plant genotype and agricultural management on the presence, nodulation capacity and genotypic diversity of rhizobia.

**Method:**

A field experiment was conducted with twelve common bean genotypes under irrigation and rain-fed conditions, both in conventional and organic management. Estimation of the number of viable rhizobia present in soils was performed before the crop establishment, whereas the crop yield, nodule number and the strain diversity of bacteria present in nodules were determined at postharvest.

**Results:**

Rainfed conditions reduced the number of nodules and of isolated bacteria and their genetic diversity, although to a lesser extent than the agrochemical inputs related to conventional management. In addition, the effect of water scarcity on the conventional management soil was greater than observed under organic conditions.

**Conclusions:**

The preservation of diversity will be a key factor to maintain crop production in the future, as problems caused by drought will be exacerbated by climate change and organic management can help to maintain the biodiversity of soil microbiota, a fundamental aspect for soil health and quality.

## 1 Introduction

Nitrogen-fixing bacteria are a widely distributed phylogenetic group of prokaryotic microorganisms that play a crucial role in the functioning of ecosystems since they are involved in the entry of nitrogen into the soils (Borges et al., 2016). These microorganisms take atmospheric nitrogen (N_2_), the most abundant component of the atmosphere, and convert it to assimilable nitrogen for plants (NH_4_^+^), using the nitrogenase enzyme. Although most species can fix nitrogen in their free-living form, some of the microorganisms need to be associated with plants, thus symbiotic associations account for 50–70% of biological nitrogen fixation (BNF) in the world (Prasuna, 2014; Simon et al., 2014).

This symbiosis provides legumes a relevant ecological advantage as well as exceptional nutritional properties. Through BNF, legumes fulfil the N requirements needed for their growth (Masson-Boivin and Sachs, 2018; Song et al., 2024). This reduces the need of synthetic N fertilizers and improves the N content of the soils, increasing their fertility and, enabling crop development in N poor soils (Aserse et al., 2020; Lindström and Mousavi, 2020). Thereby, in farming systems, legumes are often used in crop rotation, as well as green fertilizers (Araújo et al., 2015; Aserse et al., 2020). Based on their ability to colonize low-N environments and represent an alternative for saving inputs and conserving resources, the Food and Agriculture Organization of the United Nations (FAO, 2013), considers legumes as one of the most promising components of the climate smart agriculture concept.

Drought is the most severe abiotic stress in agriculture, limiting crop growth and yields, and due to climate change, drought events are expected to increase in the early years, especially in southern Europe (IPCC, 2021). It is therefore essential to seek strategies to maintain food security in a sustainable way under water-limited conditions, and the selection of drought tolerant genotypes is one of the most important goals in breeding programs.

However, in the case of legumes, several authors have suggested that selecting drought tolerant rhizobia strains could be a more determining factor in drought tolerance than selecting a drought tolerant genotype (Mhadhbi et al., 2011; Sharaf et al., 2019). Thus, the establishment of symbiotic relationships with efficient rhizobia can alleviate the effects of stress in legumes (Igiehon et al., 2021; Omari et al., 2022; Oviya et al., 2023). This is the case of common bean, where it has been observed that symbiosis with drought-tolerant rhizobia improves plant tolerance to stress as well as legume yield and quality (Steiner et al., 2020; Rodiño et al., 2021; del-Canto et al., 2023), even under field conditions (Pastor-Bueis et al., 2019; Rodiño et al., 2021).

Rhizobia, are abundant in the soil of many ecosystems and show a great diversity at the species level, as well as great variability in their symbiotic efficiency (Borges et al., 2016; Lindström and Mousavi, 2020; Guanzon et al., 2023). Unfortunately, several abiotic factors such as drought can influence the survival, functioning and diversity of soil rhizobia, and thus, legume crop productivity (Benjelloun et al., 2019; Sharaf et al., 2019; Santillana Villanueva, 2021), because few strains of rhizobia show high tolerance to water stress (Sharaf et al., 2019; Sindhu et al., 2020). In addition, strain survival and competitiveness are not correlated with their N_2_ fixation efficiency (De Souza et al., 2016; Kibido et al., 2019; da Silva et al., 2024), reducing the possibilities of establishing efficient symbiotic relationships.

Therefore, a higher diversity of legume nodulating bacteria in the soil will maximize the biological nitrogen fixation under stress conditions (Borges et al., 2016; Lindström and Mousavi, 2020; Martins et al., 2024). In this sense, greater soil microbial diversity will favour the adaptation of microbial populations to different environments, increasing the likelihood of survival of stress-tolerant microbial species and the establishment of effective symbiotic relationships (Wang and Young, 2019), contributing to greater crop resilience to stress. In addition, a greater biodiversity improves soil structure, nutrient cycling, and nutrient and water uptake, especially under drought conditions (Prudent et al., 2020).

Unfortunately, conventional agriculture related practices such as the use of herbicides and fungicides have a negative effect on the soil microbiota survival and diversity, reducing the efficiency of symbiotic relationships (Silva Neto et al., 2013; Da-Silveira-Cardillo et al., 2019; Rao et al., 2019). Additionally, the continued use of inorganic N-fertilizers causes the evolution of less-mutualistic rhizobia (Heath and Tiffin, 2007; Weese et al., 2015; Rao et al., 2019), and the decrease of nodules production (Heath et al., 2010; Regus et al., 2016). Thereby, with domestication and breeding in high-soil-N environments, the natural legume defences against less-effective rhizobia strains have been altered favouring less-cooperative rhizobia and reducing the agricultural benefits of the symbiosis (Weese et al., 2015).

Organic or sustainable farming, contrary to conventional production, promotes the biodiversity of agrosystems based on the concept that the greater biodiversity of the system, the greater health and resilience of ecosystems (Pimentel et al., 2005; Kremen and Miles, 2012; Wang and Young, 2019). The diversity of crops and their rotation system prevents the total depletion of nutrients from soils (Wolff and Killebrew, 2010; Hossain and Bakhsh, 2020) increasing soil fertility, and the diversity and activity of soil micro and macrobiotic communities (Herencia et al., 2020; Prudent et al., 2020). In this regard, studies about the abundance and diversity of rhizobia in soils, as well as the factors affecting both parameters, are of special importance to study the responsiveness of agrosystems to stresses (Omari et al., 2022).

According to different authors, the conservation of soil rhizobial diversity in agrosystems is a sustainable strategy of great interest to improve crop tolerance to stress by favouring the establishment of efficient symbiotic relationships even under water scarcity conditions (Wolff and Killebrew, 2010; Szparaga et al., 2019; Hossain and Bakhsh, 2020). In addition to this, there is interest in the search for indigenous inocula that are better adapted to local growing conditions and therefore more efficient in responding to stress conditions (del-Canto et al., 2023).

This is especially important in common bean under drought conditions as it is a very drought-sensitive crop (Embrapa, 2018; Nawaz et al., 2021) with a high frequency of inefficient symbiotic relationships (Michiels et al., 1998; Mwenda et al., 2023) that affect productivity. Considering the high nutritional value of common bean and that it is the grain legume for human consumption with the highest production worldwide (Beebe et al., 2013; FAO, 2021), the search for strategies to improve its productivity under conditions of low water availability is a challenge of great interest.

With this in mind, our hypothesis was that the type of management would have an effect on bean response to water stress, as greater microbial diversity could favour greater crop resilience. To test this, we analysed the effect of management (organic, conventional) on the production of several common bean genotype under water scarcity and how they affect the abundance, nodulation capacity and genotypic diversity of common bean rhizobia.

## 2 Materials and methods

### 2.1 Plant material

Twelve bushy genotypes of *Phaseolus vulgaris*, most of them of great economic interest in the North of Spain, were selected for the evaluation of the effect of water scarcity and management system in nodulation and rhizobia diversity. Of the twelve genotypes, four correspond to commercial genotypes and eight to locally adapted genotypes from different rainfall areas (Online resource **Supplementary Table SI.1**). The eight local genotypes, not studied to date, are traditionally grown on small family farms typically under rainfed conditions. Five of them are from the Basque Country (Northern Spain): Arrocina de Álava (AA), Amarilla de Kuartango (AK), Morada de Usánsolo (MU), Pinta Alavesa (PA) and Verde de Orbiso (VO). One is originally from Navarra (Northern Spain), Negra de Basaburua (NB), and two from Castilla y León (Central Spain), Canela de León (CL) and Riñón de León (RL). The other four are commercial genotypes commonly grown all around Spain: Cocco Blanco (CB), Lingot (L), Negrita (N), also marketed as “Frijol Negro”, and Borlotto de Vigevano (B).

### 2.2 Location and soil characterization

The trials were performed in NEIKER experimental farm located in Arkaute, Álava (Spain), between May and August of 2015. Arkaute (WGS84: 42.850254, -2.621362) is located at 532 meters above sea level and has an oceanic climate, type Cfb (temperate oceanic climate or subtropical highland climate), according to the Köppen Geiger climate classification (1900), which is, a temperate and humid climate, in transition with the Mediterranean climate (information obtained from *Euskalmet, Euskal meteorologia agentzia*). The average temperature throughout the growing season was 17.8°C, with three days having a minimum temperature less than 5°C and 6 days having a maximum temperature that exceeded 35°C. The accumulated precipitation during the experiment was 116.5 mm.

**Figure 1:**
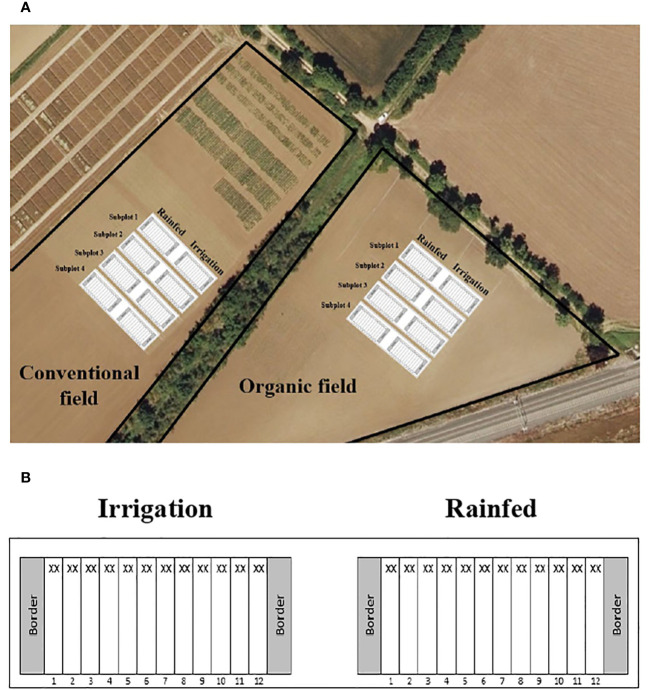
Location of the two crop fields of the study, including the experimental design scheme **(A)**, and detail the subplots **(B)**.


The field experiment was conducted under conventional and organic management. Both types of soils were catalogued according to European standards and the current legislations of the Government of Spain (MAPA, 2023), which describe and classify the type of practices in each management system. The soil of the selected plots for the study presented close locations (one contiguous to the other), similar cropping histories (rotations between cereals, potatoes, vegetables and legumes), and generally were grown under irrigated conditions (**Figure 1**). The main differences in the management history of both soils were due to agrochemical supplies. In the conventional plots, different agrochemicals (herbicides, pesticides, and chemical fertilization) were frequently applied depending on the different types of crops and the requirements of each year. In the other hand, organic plots avoided the use of synthetic chemical products. The supply of nitrogen and nutrients was provided through organic amendments, according to the Council Regulation EC No 834/2007 of 28 June 2007. The conventionally managed soil has been worked under this type of management for more than ten years, while the organically managed soil has been worked in this way, without the use of synthetic chemicals, for five years. Although common beans were frequently grown in both soils, no plot had a history of commercial inoculation with rhizobia. Therefore, all the possible rhizobium inoculums were naturally occurring.For the soil characterization, eight soil samples 20 cm deep were randomly collected from each soil (conventional and organic) and analysed at the Fraisoro Agro-environmental Laboratory (Diputación Foral de Gipuzkoa) to study their physical-chemical characteristics, according to the official methods of analysis of the Ministry of Agriculture of Spain (MAPA, 1994). The Ph, electrical conductivity in calcium sulfate (EC, µS·cm^-1^) and effective cation exchange capacity (CEC, meq·100 ml^-1^) were determined by ADAS method; the organic matter content (OM, %) was determined by the Walkley-Black method without heat input; the nitrogen (N) content (%), by Kjeldahl method; the phosphorous (P) content (mg·L^-1^), by the Olsen-Watanabe method performing an extraction in sodium bicarbonate at pH 8.5; the content of potassium (K, mg·L^-1^), calcium (Ca, mg·L^-1^) and magnesium (Mg, mg·L^-1^), by ADAS method with extraction in ammonium nitrate and subsequent reading in ICP-OES. The C/N balance was calculated based on the following formula:

C/N=(OM content/1.72)/N content

Where 1.72 is the factor of Van Bemmelen for the conversion of organic matter into Carbon. Finally, the granulometric characteristics of the soil (percentages of fine sand, coarse sand, silt, and clay) were determined according to the ISSS soil particle size fraction system.

### 2.3 Estimation of the number of viable rhizobia present in soils

The number of viable rhizobia present in the soils was estimated according to the methodology described by Hungría and Araujo (1994) with little modifications. The most probable number (MPN) of viable rhizobia is an indirect method for counting rhizobia present in soils. This method assumes that an infectious or viable rhizobia is capable of developing a nodule. While a negative result, absence of nodules, indicates the absence of infectious rhizobia. For that, before the crop establishment, soil samples were taken from both conventional and organic management. The soil samples were passed through a 4 mm sieve. Then, 10 g of processed soil was diluted in 95 mL of diluent solution (phosphate buffer, pH 7.3) in a beaker with glass pearls on a horizontal shaker at slow speed at a temperature of 25°C for 30 minutes. From this soil solution, five serial dilutions were made (1 mL of previous solution and 9 mL of diluent solution), making four replications of each one. In addition, a small aliquot of the sieved soil sample was weighed (fresh weight, FW), and after drying in the oven at 80°C for two days, the sample was reweighed (dry weight, DW) in order to calculate the humidity factor (HF) and to compare the different soils regardless of its water content with the following formula:


$$HF=\left(FW-DW\right)/{\left(DW-recipient\;tare\right)}^{-1}$$

Previously, sterilized common bean seeds of Arrocina de Álava genotype (10 min in sodium hypochlorite 1%) were germinated in opaque cultivation jars containing 500 mL of nitrogen-free Fahraeus solution (Fahraeus, 1957). The seeds were held in a funnel, connected to the nutrient solution by a filter paper wick. Then, each of the seedlings were inoculated with one of the serial solutions of soil and grown in a growth chamber (Ibercex SA, Alcalá de Henares) under controlled conditions (12 h photoperiod, light intensity 500 µmoles of photons·m^-2^·s^-1^, 20/25°C temperature and 70/60% relative humidity, night/day respectively). After three weeks, the number of plants that developed nodules (positives) and those that did not (negatives) were counted. From these values, using a mathematical formula or a table of results (Hungría and Araujo, 1994), and considering the soil humidity factor (HF), the MPN of viable rhizobia was estimated, as well as the occurrence probabilities and lower and upper limits of the 95% confidence interval.

### 2.4 Experimental design and growth conditions

Irrigated and rain-fed treatments were performed in two subplots from each selected management plot. One subplot for conventional and the other for organic management. The 12 genotypes were sown by hand at a depth of 2 cm using a randomized block design with 4 biological replicates per water regime. A distance of 5 m separated each block, which consisted of two 10 m long rows with a 0.5 m between rows and 0.2 m separation between plants within the row, achieving a stand density of 100,000 plants ha^-1^.

The water inputs were estimated controlling the irrigation time of the irrigation system (6 mm·h^-1^ flow), while the rainfall data were taken from the weather station Arkaute I (Euskalmet, 2023) located close to the experimental fields. Under rain-fed conditions, the seeds from common bean genotypes described above only received a minimum amount of water after sowing to assure the emergence and seedlings survival (12 mm). Afterwards the seedlings only received rainwater (116.5 mm). For the irrigated trials, three supplemental irrigations were supplied to plants during their growth (12 mm each). Therefore, the irrigated plots received a total contribution of 164.5 mm, while those under rainfed conditions received 128.5 mm, which is, a 22% reduction of water availability.

Under organic management, weeding control was mechanical and manual. In fields under conventional management, various herbicides were applied: preemergence herbicide Linuron 500 g·L^-1^ (Linurex ^®^ 50 SC, Adama Chile SA), at a minimum recommended dose of 0.8 L·ha^-1^; and post-emergence herbicide, pendimethalin 45.5% (Stomp^®^ Aqua, BASF), at a minimum recommended dose of 2 L·ha^-1^, at the V3 development stage, plants with the first trifoliate leaf (Fernández et al., 1986).

### 2.5 Nodule sampling

In the pre-flowering stage, R5 (Fernández et al., 1986), the nodules from three randomly selected plants from each experimental plots were collected, with four replicates of each, i.e. 12 nodules were harvested from each experimental condition (three plants of 12 genotypes, grown under two different water regimes in two different agricultural managements, with four replications) a total of 576 plants. Using a shovel, and measuring 20 cm from the center of the plat, the root system was excavated until a depth of 26 cm totalling a soil volume of 10.4 L per plant, similarly to Somasegaran and Hoben (1994), to ensure the harvest of practically the entire root system. Once in the laboratory, whole plants were carefully removed from the soil to obtain roots and nodules, and adhering soil was removed from roots by careful shaking. Then, the removed soil was carefully examined to recover any nodules left in the soil. Harvested nodules were washed in water and gently dried with paper. The nodules were counted and desiccated in bottles with silica gel at 4°C to preserve and use them in future research.

### 2.6 Yield quantification

At harvest, all plants from each block were counted, collected individually, dried, threshed, and cleaned separately to quantify the yield (Kg·ha^-1^). Four biological replicates of each experimental conditions.

### 2.7 Endosymbiont isolation

The use of common bean “trap plants” grown in local field under rainfed conditions, could guarantee that isolated rhizobia have a certain competitiveness and possibly tolerance to water stress. Therefore, once the four most productive genotypes under rainfed conditions and a less productive one were selected, the bacteria present in their nodules were extracted from eight nodules randomly picked from each plot according to Sanz-Sáez et al. (2015). That is, 8 nodules per experimental condition, a total of 160 nodules (8 nodules of 5 genotypes grown under rainfed and irrigation conditions in two agricultural management systems). The nodules were first rehydrated by immersion in sterile distilled water for 2-4 h and surface-sterilized by immersing them in 70% ethanol (5 sec), and then in 50% sodium hypochlorite (5 min). Later, they were rinsed several times with sterile distilled water to remove the bleach residues. The nodules were crushed in a petri dish and 10-20 μL of autoclaved distilled water was added on the medulla of the nodule, aspirating, and expiring several times with the micro pipette to collect the bacteria (Somasegaran and Hoben, 1994; Howieson and Dilworth, 2016) and placing on a plate with solid TY medium (Beringer, 1974) to be grown at a constant temperature of 29°C.

Once the bacteria had grown, a single colony was chosen randomly and replated to a new solid TY plate for purification of rhizobia isolates. This operation was repeated for all observed colonies of different morphology and appearance from each plate. Using this method, 368 total isolates were obtained. These pure cultures were preserved at 4°C and were then transferred to 50% glycerol in TY, to preserve them at -80°C, in order to use them in future trials.

### 2.8 Isolated strain diversity by BOX+REP polymerase chain reaction genomic fingerprinting

Consensus sequences such as repetitive extragenetic palindromic sequences (REP), enterobacterial repetitive intergenic consensus (ERIC) and BOX elements, related to repetitive and conservative elements diffused in DNA, have been extensively used for rhizobial strain identification due to its ease, quickness, and high discriminatory power at infraspecific level (Kaschuk et al., 2006; Borges et al., 2016; Benjelloun et al., 2019). In our study, we used BOX and REP-PCR since the application of both PCR methods increases the accuracy when compared with only one PCR (Olive and Bean, 1999). The DNA extraction was performed according to Heath and Tiffin (2009) with modifications: 104 μL of liquid medium TY (Beringer, 1974) incubated with bacteria during 24-48 h at 28°C was centrifuged for 5 min at 16000 g and the pellet containing the cells was washed 3 times with 1 M NaCl and once with 100% ethanol and dried before extracting DNA. Then, the cells were re-suspended in 104 µL of sterile milli-Q water and homogenizing gently with the micropipette. The suspension was centrifuged 10 min at 16000 g and the aqueous phase was removed. For the DNA extraction, 30 µL of 10 mM tris-HCL (pH 8) and 1 µL of 20 mg·mL^-1^ protein kinase K enzyme (Invitrogen, Carisbad, CA) was added to the pellet and homogenized again with the micropipette and vortex. Then, 30 µL were transferred to a 0.2 mL PCR tube, and 55°C was applied for 4 h in order to let the protein kinase K to perform its function. After that it was deactivated by heating the solution at 95°C for 10 min. DNA samples were quantified using a NanoDrop spectrophotometer (Thermo scientific, Wilmington, DE, USA) and diluted to 25 ng μL^-1^. The DNA extraction was stored at 4°C.

BOX-PCR was performed according to the method described by Kaschuk et al., 2006 with modifications, using the BOX A1R primer (5′-CTACGG CAAGGCGACGCTGACG-3′; Versalovic et al., 1991). REP-PCR was performed according to the method described by Versalovic et al., 1991 with modifications, using the primers REP-1 (5´-IIIICGICGICATCIGGC-3´) and REP-2 (5´-ICGICTTATCIGGCCT AC-3´). Each PCR reaction was performed in a final volume of 10 μL containing: dNTPs 0.3 μL (10 mM); reaction buffer 1μL (10x BioLabs, New England); primer 0.5 μL (10 mM), for two primers in REP; Taq DNA polymerase 0.08 μL (5 Um·L^-1^); Betaine 0.5 μL (5 mM), DNA 1.5 μL; sterile milli-Q water to complete the volume. The amplification program was performed in a thermocycler S1000™ (Bio-Rad Laboratories, Inc.), applying an initial denaturing step (95°C, 7 min) with 30 cycles of denaturation (95°C, 1 min), annealing (53°C, 8 min for BOX-PCR and 40°C; 8 min for REP-PCR) and extension (65°C, 8 min); and a final extension cycle (65°C, 16 min). The PCR products were separated by horizontal electrophoresis on a 1.5% agarose gel (EEO-Mr<0.15) in TBE buffer (0.5x) at 90 V for 5 h, using a 1 Kb DNA marker (BioLabs, New England). The gels were stained with ethidium bromide, and visualized under UV light using a benchtop UV transilluminator (Bio-Doc-It™ UVP Imaging System).

### 2.9 Statistical analysis

The diversity of strains was analysed from the images of the gels (PCR fingerprints) using the free software GelJ v.2.0 (Heras et al., 2015) and transformed into a binary matrix. After analysing a large number of samples, the most consistent bands were selected, with 70% or more percentage of appearance. BOX and REP-PCR data were combined for each isolate.

From this BOX+REP binary matrix, Python software was used to build similarity matrices. Using the Jaccard coefficient and applying the UPGMA algorithm unweighted pair-group method with arithmetic mean (Sokal, 1973), the dendrogram and diversity indexes (Shannon and Weaver, 1949), richness (Margalef, 1958) and evenness (Pielou, 1977) were obtained. The graphic representations of the dendrograms were made with the free software iTOL (Interactive tree of Life, Leunic and Bork, 2019). Through these analyses, the different isolates were grouped according to the degree of similarity of their PCR fingerprints in different clusters. The number of clusters also indicates the strain diversity or diversity at infraspecific level. The number of clusters and their bootstrapping was calculated at 70% similarity. Due to the observed high variability, to obtain larger clusters, 35% similarity was also used (Grange and Hungria, 2004).

The nodule number and yield was analysed using the statistical package SPSS Statistics 24.0 (IBM Corporation, Armonk, NY, USA). The normality of the non-standardized residuals of the data was studied using the Shapiro-Wilk test and the homoscedasticity of the variance was studied with the Levenne test. As the water availability treatment was not randomized and organic and conventional management soils were separated, the behaviour of the genotypes was studied in the four experimental conditions: irrigated conventional management; rainfed conventional management; irrigated organic management; and rainfed organic management as it has been performed previously by Sanz-Sáez et al. (2019). For this, a one-way analysis of variance (ANOVA) was performed with genotypes as factor and replicates as random effect. When the genotype effect was significant, least square means *post-hoc* test was performed to compare means (Tukey or Kruskal Wallis). The management effect was also analysed separating the data according to the water regime through one-way analysis of variance (ANOVA), with agricultural management as factor and replicates as random effect. The graphic representations of the nodule number were made with SigmaPlot 15 (Systat Software, Inc.).

As one of the objectives of this research was to investigate the effect of water scarcity over the nodulation, the two management systems were treated as two different locations (environments) and the effect of water scarcity was analysed for each location separately, despite not being randomized. One-way ANOVA with water availability was treated as factor and replicates as random effect.

## 3 Results

### 3.1 Soil characterization

The properties and characteristics of soils cultivated under organic and conventional management were similar (**Table 1**). Both were clay-loam soils with a very light salinity, had a basic pH typical of limestone soils, contained an optimal carbon balance, and contained adequate nitrogen and phosphorus content with a medium magnesium content. However, both soils differed in their organic matter, magnesium and potassium content all being higher in the organic management soil, while higher calcium content occurred in the conventional management field.

**Table 1:** Physical-chemical characteristics of conventional and organic agricultural soils of the experimental fields.

	Conventional	Organic
pH	8.30	8.45
Electrical Conductivity (μS·cm^-1^)	1900	1700
C/N balance	3.44	7.62
Organic Matter (%)	1.12	1.43
Nitrogen (%)	0.19	0.20
Phosphorous (mg·L^-1^)	42.81	46.75
Potasium (mg·L^-1^)	164.0	340.00
Magnesium (mg·L^-1^)	85.20	127.20
Calcium (mg·L^-1^)	6850	6284
Sodium (mg·L^-1^)	34.50	29.90
Efective CEC (meq/100 mL)	20.76	16.81
Fine sand (0.2-0.02 mm, %)	34.34	48.50
Coarse sand (0.2-2 mm, %)	7.81	3.97
% Silt (0.02-0.002 mm, %)	24.53	29.40
% Clay (<0.002 mm, %)	33.32	18.10
Texture clasification	Clay-Loam	Clay-Loam

### 3.2 Estimation of the number of viable rhizobia present in soils

The estimation of rhizobia cells existing in the sampled soils before sowing and the establishment of water treatment are shown in **Table 2**. The MPN shows the number of live rhizobia cells present per unit of volume in the matrix solution taking into consideration the soil humidity correction factor which allows for comparisons of the different soils regardless of water content. The MPN values were more than 20-fold higher in the organic (21.102) than in the conventional (0.609) management soil (**Table 2**).

**Table 2:** Most probable number of viable rhizobia in organic and conventional plots before sowing: HF, soil humidity factor; MPN, most probable number of viable rhizobia cells present in the soil matrix solution corrected with HF; P (%), probability of the combination occurrence if the experiment is repeated an infinite number of times with the same matrix solution; CImin and CImax, lower and upper limits of the 95% confidence interval.

	HF	MPN	P (%)	CI_min_	CI_max_
**Conventional**	1.0	**0.6**	28.2	0.2	2.3
**Organic**	1.2	**21.1**	0.2	9.3	52.8

### 3.3 Effect of water stress, agricultural management and legume genotypes on nodulation

The infection capacity of rhizobia, showed as nodule number per plant, was almost 58.25% lower, on average, in conventional management than in organic management (**Table 3**). The number of nodules was also reduced by low water availability in both management systems although its effect was less evident than that of the management itself. Although there was not management by water availability interaction, rainfed conditions seemed to decrease the rhizobia infection capacity more in conventional (24.56%) than in organic management (20.30%) (**Table 3**).

**Table 3:** Mean values ( ± SE) and ANOVA results (p-value) of nodule number per plant of different common bean plants grown under different water availability (WA) conditions: irrigated and rainfed and in different management systems (M), conventional and organic.

Management	WA	Nodule number per plant
Conventional	Irrigation	6.80 ± 0.89 B
	Rainfed	5.13 ± 0.93 b
	**Mean**	**6.37 ± 0.75**
Organic	Irrigation	15.41 ± 0.95 A
	Rainfed	12.28 ± 1.11 a
	**Mean**	**15.26 ± 1.05**
ANOVA RESULTS		
Factors	P-value	
M	***	
WA	**	
M*WA	NS	

Capital letters were used to compare the water availability treatments in conventional management and lowercase letters in organic management (*p<0.05; **p<0.01 and ***p<0.001; NS, non-significant).

**Figure 2:**
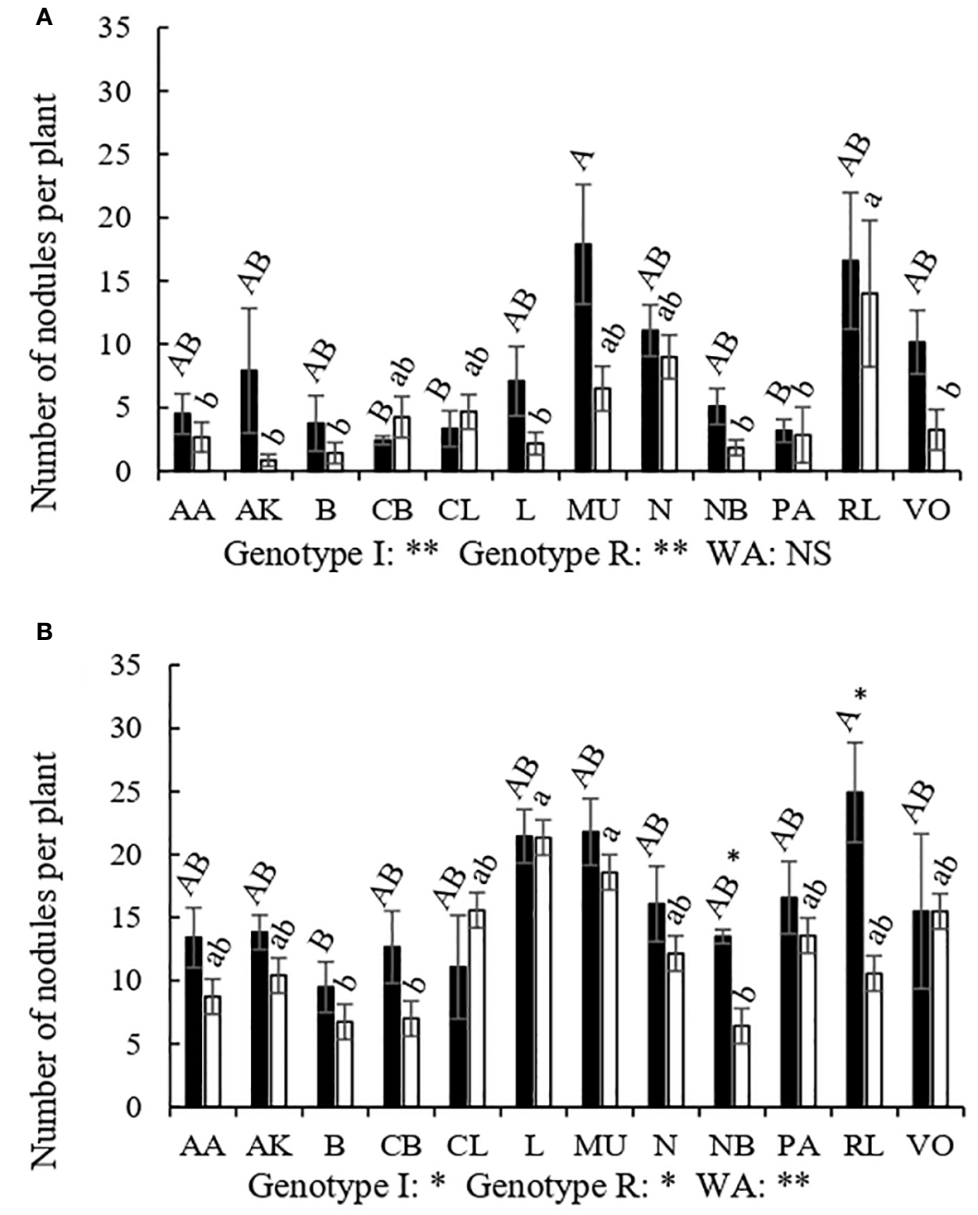
Number of nodules per plant of different genotypes of *Phaseolus vulgaris* (AA, Arrocina de Álava; AK, Amarilla de Kuartango; B, Borlotto; CB, Coco blanco; CL, Canela de León; L, Lingot; MU, Morada de Usansolo; N, Negrita; NB, Negra de Basaburua; PA, Pinta alavesa; RL, Riñón de León; y VO, Verde de Orbiso), under different water availability (WA) treatments in the field: irrigation (dark) and rainfed (light); both in conventional **(A)** and organic management **(B)**. Bars indicate the mean of each genotype for number of nodules. Different capital letters designate significantly different genotype means under irrigated conditions while lower case letters designate significant different genotype means for the rainfed treatment according to LSD *post-hoc* analysis. Asterisks withing the figures were used to show the effect of water in each genotype separately. Genotype I illustrate the p-value of the genotype effect under irrigated conditions; Genotype R illustrates the p-value of the genotype effect under rainfed conditions; WA, indicated the effect of water availability factor in each management (*p<0.05; **p<0.01 and ***p<0.001; NS, non-significant).

The nodulation capacity also varied depending on the genotypes although all of them showed higher nodulation capacity in organic than in conventional management (**Figure 2**). The genotypes RL and MU showed a greater number of nodules under irrigation in both management systems, while CB, PA and CL showed the least in the conventional management, and B showed the least in the organic management field. Reduced water availability only decreased nodule number in RL and NB genotypes in the organic plots, whereas in conventional plots no significant effect of rainfed conditions was observed in any of the genotypes tested. RL was by far the genotype that developed a greater number of nodules under rainfed conditions in conventional management (**Figure 2**) but was more affected by water availability in organic management. Under organic management, the MU and L cultivars were those that showed greater nodulation under rainfed conditions, unlike CB, B and NB that were the less nodulated genotypes. The B and NB genotypes also showed less nodules in conventional management under rainfed conditions. Therefore, the genotypes that most times showed the highest nodulation capacity were the RL and MU, while the B and NB were the ones showing the lowest nodulation capacity.

### 3.4 Yield quantification

The yields obtained in conventional management were higher than those obtained with organic management. However, while rainfed conditions considerably reduced common bean production under conventional management, water scarcity did not significantly reduce organic bean production (**Table 4**).

**Table 4:** Mean values ( ± SE) and ANOVA results (p-value) of the effect of water availability (WA) and genotype (G) on common bean yield (kg·ha^-1^). both in conventional and organic management.

	Conventional yield (kg·ha-1)			Organic yield (kg·ha-1)	
	Irrigation	2746.2 ± 155 a		Irrigation	1649.5 ± 135.2 a
	Rainfed	2350.7 ± 111.2 b		Rainfed	1451 ± 87 a
	ANOVA RESULTS			ANOVA RESULTS	
	Factors	P-value		Factors	P-value
	WA	*		WA	NS
	Conventional yield (kg·ha^-1^)			Organic yield (kg·ha^-1^)	
Gen.	Irrigation	Rainfed	Gen.	Irrigation	Rainfed
AA	3807.8 ± 237.5	2679.5 ± 146.1 abc	AA	3107.3 ± 505.2 a	2395.7 ± 444.7 a
AK	2232.8 ± 450.8	1882.1 ± 381.3 bc	AK	1031.7 ± 158 b	1052.9 ± 153.9 b
B	2121.9 ± 379.7	1837.6 ± 246.3 c	B	1156.1 ± 163.5 b	951.4 ± 117.1 b
CB	2288.4 ± 291.1	2174.2 ± 334.2 bc	CB	867.2 ± 376.5 b	1139.7 ± 256.8 b
CL	2358.6 ± 377.5	1892.9 ± 296.9 bc	CL	1442.6 ± 196.3 ab	1189.7 ± 201.8 b
L	3462.8 ± 395.1	2092.7 ± 144.1 bc	L	1444.7 ± 208.2 ab	1750.8 ± 111.7 ab
N	2850.4 ± 686.5	2981.2 ± 398 ab	N	2179.6 ± 229.9 ab	1881.9 ± 344.4 ab
NB	3216.6 ± 416.7	2631.6 ± 251.8 abc	NB	1910.7 ± 381.7 ab	1562.7 ± 142.6 ab
PA	2890.8 ± 670.3	2424 ± 219.7 abc	PA	1384.7 ± 406.7 ab	1380 ± 242.5 ab
RL	3926.9 ± 343.2	3285.9 ± 509.7 a	RL	1884.5 ± 480.2 ab	1610.8 ± 42.9 ab
VO	2205.3 ± 254.9	2174.4 ± 215.4 bc	VO	1794.5 ± 658.1 ab	1160.2 ± 173.4 b
ANOVA RESULTS			ANOVA RESULTS		
Factors	P-value	P-value	Factors	P-value	P-value
G	NS	*	G	*	**

AA, Arrocina de Álava; AK, Amarilla de Kuartango; B, Borlotto de Vigevano; CB, Cocco Blanco; CL, Canela de León; L, Lingot; MU, Morada de Usansolo; N, Negrita; NB, Negra de Basaburua; PA, Pinta alavesa; RL, Riñón de León; and VO, Verde de Orbiso (*p<0.05; **p<0.01; NS, non-significant).

On the other hand, it was observed that AA, N, NB and RL were the genotypes that ranked as most productive under different experimental conditions, also under rainfed conditions, while AK was one of the least productive (**Table 4**). Thus, those were the genotypes selected to study the diversity of the bacteria strains inside their nodules.

### 3.5 Isolated strain diversity by BOX+REP PCR genomic fingerprinting

From the 368 bacteria isolated from nodules of plants grown under different water availability in both organic and conventional management, BOX+REP PCR fingerprinting were obtained on 320 isolates, since some strains did not amplify with the primers used similarly to other literature (Judd et al., 1993; Mostasso et al., 2002; Kaschuk et al., 2006).

**Figure 3:**
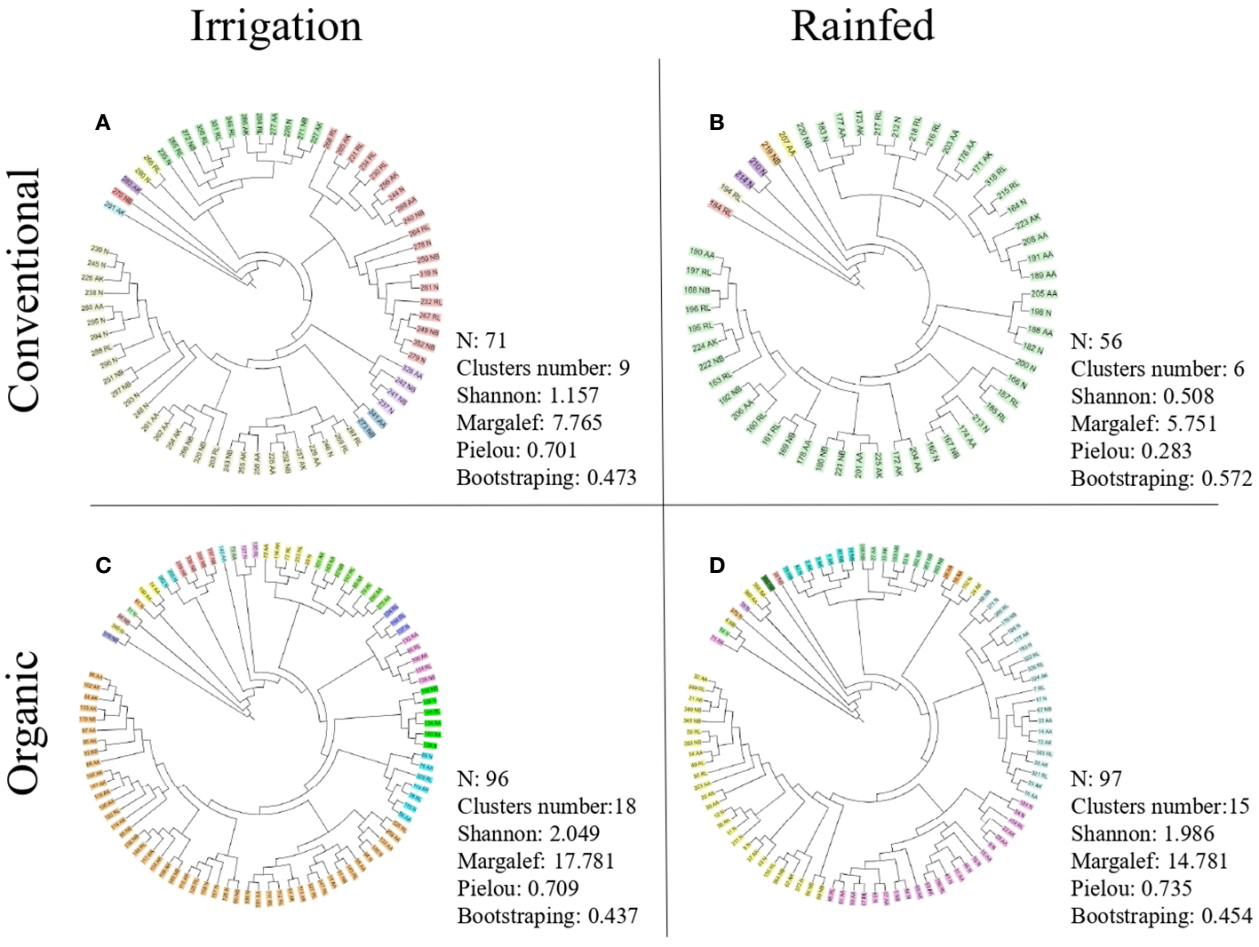
Dendrogram constructed from the BOX+REP PCR genomic fingerprinting. Number of isolated bacteria (N), number of clusters (represented graphically with different colours), diversity indices (Shannon, Margalef and Pielou) and cluster bootstraping obtained at a 35% similarity level in: conventional management under irrigation conditions **(A)**; conventional management under rainfed conditions **(B)**; organic management under irrigation conditions **(C)**; and organic management under rainfed conditions **(D)**.

The genomic fingerprinting showed a high level of genetic diversity among the strains, considering a similarity of 70% in the clustering analysis, confirmed by the low final levels of similarity (Online Resource, **Supplementary Table SI.2**). With this level of similarity (70%) the vast majority of the generated clusters in the different experimental conditions were composed of a single strain and the values obtained from bootstrapping were generally greater than 0.60, thus, can be considered stable groups (Jain and Moreau, 1987; Kerr and Churchill, 2001). To obtain larger clusters, formed by a greater number of strains, the isolated strains were also grouped at a 35% similarity level (**Figure 3**, **Tables 5**, **6**). Thus, larger clusters were obtained and formed by several strains, although bootstrap values remained below 0.60.

**Table 5:** Number of isolated bacteria, average stability of clusters and mean values ( ± SE) and ANOVA results (p-value) of strain diversity indices (Shanon, Margalef and Pielou) and number of obtained clusters at a similarity level of 35% according to agricultural management (conventional and organic) and water availability (I, irrigated; and R, rainfed) (*p<0.05; **p<0.01 and ***p<0.001; NS, non-significant).

			Isolated bacteria	Cluster stability (bootstraping)	Diversity (Shannon´s index)	Richness (Margalef´s index)	Evenness (Pielou´s index)	Number of clusters
**Conventional**			127	0.51	1.27 ± 0.12 b	11.79 ± 0.32 b	0.51 ± 0.05 a	12 ± 0.30 b
**Organic**			193	0.46	2.17 ± 0.152 a	14.81 ± 0.70 a	0.69 ± 0.04 a	23 ± 0.69 a
**Irrigation**			167	0.44	2.13 ± 0,15 a	20.80 ± 0.77 a	0.70 ± 0.04 a	21 ± 0.77 a
**Rainfed**			153	0.44	1.79 ± 0.16 a	17.80 ± 0.56 a	0.62 ± 0.05 a	18 ± 0.54 a
**Conventional**		**I**	71	0.47	1.16 ± 0.115 a	7.76 ± 0.52 a	0.7 ± 0.06 a	8 ± 0.51 a
		**R**	56	0.57	0.51 ± 0.12 a	5.75 ± 0.40 a	0.28 ± 0.06 a	6 ± 0.37 a
**Organic**		**I**	96	0.44	2.05 ± 0.27 a	17.78 ± 1.24 a	0.71 ± 0.07 a	18 ± 1.24 a
		**R**	97	0.45	1.98 ± 0.17 a	14.78 ± 0.76 a	0.73 ± 0.05 a	15 ± 0.75 a
				ANOVA RESULTS				
				Factors	P-value	
				M	**	**	NS	**
				WA	NS	NS	NS	NS
				M*WA	NS	NS	NS	NS

**Table 6:** Number of isolated bacteria, strain diversity indices (Shanon, Margalef and Pielou) and number, structure and average stability of clusters obtained at a similarity level of 35% according to agricultural management (C, conventional; O, organic), water availability (I, irrigated; R, rainfed), and genotype (AA, Arrocina de Álava; AK, Amarilla de Kuartango; N, Negrita; NB, Negrita de Basaburua; RL, Riñon de Leon).

			Isolated bacteria	Diversity (Shannon´s index)	Richness (Margalef´s index)	Evenness (Pielou´s index)	Number of clusters	Cluster stability (bootstraping)
AA	C	I	10	1.566	1.566	0.881	2	0.495
		R	15	0.720	3.631	0.519	4	0.409
	O	I	21	2.001	8.672	0.911	9	0.529
		R	19	1.979	4.660	0.931	5	0.473
		**Total**	**65**	**1.966**	**10.768**	**0.820**	**11**	**0.404**
AK	C	I	10	1.089	3.566	0.571	4	0.594
		R	6	0.451	1.442	0.650	2	0.562
	O	I	20	0.613	2.666	0.558	3	0.612
		R	18	1.132	3.654	0.817	4	0.549
		**Total**	**54**	**0.884**	**5.749**	**0.493**	**6**	**0.494**
N	C	I	18	0.937	2.654	0.853	3	0.482
		R	11	1.121	3.583	0.809	4	0.518
	O	I	18	2.000	8.654	0.910	9	0.562
		R	24	1.831	7.685	0.881	8	0.815
		**Total**	**71**	**1.735**	**12.765**	**0.676**	**13**	**0.488**
NB	C	I	16	1.180	3.639	0.851	4	0.516
		R	9	0.684	2.545	0.622	3	0.550
	O	I	16	1.581	5.639	0.882	6	0.543
		R	21	1.234	5.672	0.689	6	0.590
		**Total**	**62**	**1.527**	**11.758**	**0.615**	**12**	**0.472**
RL	C	I	17	1.401	4.647	0.871	5	0.552
		R	15	0.485	2.631	0.442	3	0.621
	O	I	21	0.996	3.672	0.719	4	0.461
		R	15	1.323	3.631	0.954	4	0.499
		**Total**	**68**	**1.602**	**9.763**	**0.696**	**10**	**0.441**

The row labeled **Total** shows the data obtained for each genotype without considering the different experimental conditions.

The number of isolated bacteria (Online resource, **Supplementary Table SI.2**, **Supplementary Table SI.3**), clusters, diversity (Shannon, H_0_), richness (Margalef, R_1_) and evenness (Pielou, E_1_) of strains were greater in the nodules of the plants grown under organic management than under conventional management, regardless of the considered similarity level (**Tables 5**, **6**, for 35% of similarity and Online Resource, **Supplementary Table SI.2**, **Supplementary SI.3**, for 70% of similarity). Rainfed conditions also caused a drop, although not statistically significant, in the diversity indices (Shannon, Margalef and Pielou), number of isolates and obtained clusters, at both levels of similarity (**Table 5** and Online resource, **Supplementary Table SI.2**). This can be observed when analysing both managements separately based on water availability (**Figure 3**). Nodules of plants grown in organic soils always showed higher strain genetic diversity (diversity indices and number of clusters), even under rainfed conditions, with respect to the most favourable conditions (irrigation) of conventional management (**Figure 3**, **Table 5** and Online resource **Supplementary Table SI.2**).

In conventional management under irrigated conditions, 71 bacteria were isolated and grouped into 9 clusters, two of which represent 67.6% of the strains. Under rainfed conventional management, the isolated bacteria were reduced to 56 bacteria and 89.3% of them were grouped into one large cluster (**Figure 3**). Nevertheless, under organic management and irrigation conditions, 96 bacteria were isolated and grouped into 18 clusters, with the largest cluster representing 46.9% of the isolated bacteria. Under rainfed organic conditions, 97 strains were isolated and grouped into 15 clusters, three of which represented 71.1% of the isolated bacteria. Rainfed conditions reduced the number of isolated bacteria strains by 21.1% under conventional management but not in organic management soil. The genetic diversity of strains (number of clusters) was reduced by 33.3% in conventional management and by 16.7% in organic management due to low water availability (**Figure 3**).

Finally, when analysing the different genotypes (**Table 6**), AK showed the lowest number of isolated bacteria (54), the lowest indexes of genetic diversity and the lowest number of clusters. On the other hand, N and NB, showed the highest indexes of genetic diversity and a greater number of clusters (13 and 12 respectively). N was the genotype from which the greatest number of bacteria were isolated (71).

## 4 Discussion

The great diversity that rhizobia show at the species level is essential for *Phaseolus vulgaris* to stablish efficient symbiosis and maintain crop productivity in a sustainable way, especially under drought conditions (Lindström and Mousavi, 2020; Guanzon et al., 2023; da Silva et al., 2024). Rhizobia can play a more important role in the resistance to stress of the symbiosis relationship than the genotype of plant (Mhadhbi et al., 2011; Sharaf et al., 2019). Unfortunately, the symbiotic efficiency of rhizobia is highly variable and, on many occasions, establish inefficient symbiotic relationships with common bean having a negative impact on productivity (Michiels et al., 1998; Armenta-Borjóquez et al., 2016; Mwenda et al., 2023). Thus, greater microbial diversity in soils and the better the response of crops to stress will increase the probability of appearance of efficient stress-tolerant microbial species (Wang and Young, 2019) that can maximize biological nitrogen fixation under such conditions (Borges et al., 2016; Lindström and Mousavi, 2020; Liyanage et al., 2023).

The BOX and REP-PCR fingerprinting analysis showed a high level of genetic diversity among the bacteria isolated from nodules of all the sampled plants grown under the different agronomic conditions. These data confirm the great promiscuity of the common bean plants which are capable of nodulating with many different bacteria. Our results are comparable to those of other studies using similar techniques, in which most of the groups, clustered at 70% similarity, were formed just by one or two strains (Grange and Hungria, 2004; Chibeba et al., 2020; Odori et al., 2020).

In most clusters, the values obtained from bootstrapping were close to 0.60 at 70% similarity and even lower values were obtained at 35% of. These low bootstrap values are usually obtained in these type of dendrograms (Aulakh et al., 2020) due to both, the type of data (they are not related sequences), and to the large number of operational taxonomic units (OTUs, bacterial strains), which contribute to lower the value of the bootstraps (Efron and Tibshirani, 1993). However, despite the low bootstrap values, this technique is considered a powerful tool to detect strain diversity (Alberton et al., 2006; Kaschuk et al., 2006; Costa et al., 2018).

Water scarcity provokes reduction of nodule number per common bean plant (Berny-Mier et al., 2019; Aserse et al., 2020; Prudent et al., 2020). In this study, a reduction of only 22% in water availability affected the nodulation efficiency, reducing the number of nodules per plant and the number of isolated bacteria per nodule. However, it did not significantly affect the strain genetic diversity, although there was a tendency to decrease under water scarcity conditions.

Water scarcity is one of the factors that most affect the rhizobia survival in their free-life phase (Hungria et al., 2000; Sindhu et al., 2020; Santillana Villanueva, 2021) and the nodulation process, affecting chemotaxis, initiation, formation and development of nodules (Sindhu et al., 2020; Viti et al., 2021; Omari et al., 2022) thus reducing the number of nodules. In fact, under sufficiently prolonged or severe drought conditions the nodule formation can be completely inhibited (Gerosa-Ramos et al., 2003). In drought-affected soils, the decrease of bacterial diversity and nodulation capacity also translates into a lower diversity of the nodule bacteriome, reducing the number of isolated bacteria and the genetic diversity of detected strains. Similar effects were described in soybean (Sharaf et al., 2019) and common bean (Cytryn et al., 2007).

The reduction of the bacterial diversity parameters due to lower water availability was observed in both managements, however, the effect was slightly higher under conventional management than that observed under organic conditions. In the latter, water scarcity had no effect on yield and higher nodulation, higher number of isolated strains and greater genetic diversity of strains were recorded even under rainfed conditions. These results would demonstrate that organic soil´s microbiota reflect wider environmental adaptation and superior competitive ability (Yan et al., 2017; Costa et al., 2018) and would confirm that these soils have greater resilience to adverse conditions such as water stress via greater microbial abundance and diversity (Borges et al., 2016; Lindström and Mousavi, 2020). As some authors suggest, the maintenance of the soil microbial genetic biodiversity is of great importance because it provides a major buffering capacity of the soil (Loreau et al., 2001) and it is related to soil health and quality as well as agricultural sustainability (Kaschuk et al., 2006).

Conventional plots showed lower abundance and diversity in the soil microbiome even before sowing and under irrigated conditions. This would be related to the practices and agrochemicals used in conventional agriculture (Rao et al., 2019), since the two sampled agricultural soils, conventional and organic, had similar edapho-climatic conditions and both presented similar cropping histories and soil characteristics.

The soil of conventional management plots was subjected to years of agrochemicals and N-fertilization inputs. Mineral fertilization reduces plant nodulation (Heath et al., 2010; Regus et al., 2016) and symbiotic efficiency (Heath and Tiffin, 2007; Weese et al., 2015; Gordon et al., 2016; Rao et al., 2019) because it is energetically cheaper for plants to reduce ammonium and nitrate than to fix N_2_. This leads to a reduction on the presence and diversity of rhizobia over time (Moreira et al., 2006; Da-Silveira-Cardillo et al., 2019). In addition, during the experiment, two doses of herbicides, Pedimethalin and one of Linuron, were applied to conventional plots. There is quite a consensus on the negative effect of herbicides on bacterial diversity. Coinciding with our observations, García-Garijo et al (2014) detected that Imazamox drastically affected the nodulation and biological nitrogen fixation of common beans, and Darine et al. (2015) reported that Fusilade herbicide causes a decline in richness and structure of soil bacterial communities, mainly at the rhizosphere level. Furthermore, although the literature contains little information on the effects of pesticides on legume-rhizobia signal exchange, some *in vitro* work with 30 different pesticides showed that *Sinorhizobium meliloti* NodD was affected resulting in delayed nodulation and reduced biological nitrogen fixation in *Medicago sativa* (Fox et al., 2001).

Consequently, the use of agrochemicals leads to a loss of abundance and diversity in the soil microbiome (Rao et al., 2019), as well as of rhizobia (Silva Neto et al., 2013; Weese et al., 2015), reducing plant nodulation and genetic diversity of strains. On the contrary, organic amendments, such as manure, a common practice in organic management, usually increase the abundance and diversity of microorganisms and rhizobia (Rao et al., 2019; Li et al., 2022), promoting a greater plant nodulation (Herencia et al., 2020), as in our results.

This negative effect of agrochemicals on common bean symbiosis was even more drastic than that produced by a reduction of 22% in water availability. In fact, the use of agrochemicals reduced nodulation, the number of isolated bacteria and the genetic diversity of nodule bacteriome strains, while water availability only reduced nodulation, and no significant effect on strain diversity was observed.

Although in organic management no effect of water scarcity was observed in yield, confirming a greater resilience to water stress (Borges et al., 2016; Lindström and Mousavi, 2020), the yields obtained in this management were lower than in conventional management, probably due to the difficulty of weed control, since the physical and chemical conditions were not very different in both fields. Therefore, it is evident the need to study different strategies to improve crop productivity in organic management to avoid yield losses, such us a better weed control, one of the prime causes of yield losses in organic management (Entz et al., 2001; Posner et al., 2008).

Finally, we also detected an effect of plant genotype on nodule number, as it has been observed in several publications (Berny-Mier et al., 2019; Goyal et al., 2021; Omari et al., 2022), although without interaction with the rest of the studied factors. In addition, we detected differences in the diversity of isolated strains across genotypes, as it was observed in soybeans (Sharaf et al., 2019) and in common bean (Shamseldin and Velázquez, 2020). Although some authors have not found effect of common bean genotype on rhizobial diversity (Grange and Hungria, 2004), other researchers have reported that the legume plants control the nodulation process (Ferguson et al., 2019), and the involvement of several *Phaseolus vulgaris* genes in strain-specific selection (Rípodas et al., 2019).

RL and MU were the genotypes that showed more nodules in both management practices, also under rainfed conditions. RL was also the second genotype with a higher number of strains, after the N genotype. On the contrary, NB, was the least nodulated genotype, but showed great strain diversity and was one of the most productive genotypes under limited water conditions. On the other hand, AK, a genotype selected as the least productive, showed few nodules and low number of isolated bacteria, with less strain diversity under rainfed conditions. These results agree with the findings of other authors (Aserse et al., 2020) that suggest that the number of nodules is not always related to biological nitrogen fixation efficiency (Zurdo-Piñeiro et al., 2009; Flores-Félix et al., 2019; Shamseldin and Velázquez, 2020). However, the number of nodules gives an idea of the abundance and/or the viability of the rhizobia present in soils, and it has also been stated that more drought-tolerant strains show higher nodulation capacity under drought conditions (Aulakh et al., 2020; Omari et al., 2022; del-Canto et al., 2023).

The results show that the rhizobia isolated in this work, are efficient inocula able to nodulate under rainfed conditions as was demonstrated with some of them (del-Canto et al., 2023) and it would be interesting to test their efficiency under field conditions, following successful formulations such as those described by Pastor-Bueis et al. (2019).

## 5 Conclusion

Rainfed conditions reduced the number of nodules per plant and the number of isolated bacteria, however, the use of agrochemicals products related to conventional management had a greater negative effect than that observed by a reduction of 22% in water availability, and also affected the strain genetic diversity of the nodule bacteriome. In addition, while water limitation did not have an effect on the organic management yield, it was reduced in conventional management. Consequently, the effect of rainfed conditions on the conventional management soil was greater than observed under organic conditions.

These results would confirm that the use of agrochemicals leads to a loss of rhizobia abundance and diversity while organic management practices maintains higher values of rhizobia abundance, nodulation and diversity, even under rainfed conditions. This maintenance of diversity will be a key factor in the future, as problems caused by drought will be exacerbated by climate change. Maintaining microbial diversity implies broader environmental adaptation, superior competitive ability and greater resilience to adverse conditions. Therefore, it is necessary to develop sustainable and environmentally friendly agricultural systems, free of agrochemicals that allows for maintaining or even increasing the biodiversity of soil microbiota, a fundamental aspect for soil health and quality.

## Data availability statement

The raw data supporting the conclusions of this article will be made available by the authors, without undue reservation.

## Author contributions

AD: Data curation, Formal analysis, Investigation, Methodology, Visualization, Writing – original draft, Writing – review & editing. AS: Conceptualization, Data curation, Formal analysis, Supervision, Writing – review & editing. KH: Conceptualization, Resources, Supervision, Writing – review & editing. MG: Investigation, Methodology, Writing – review & editing. JH: Software, Visualization, Writing – review & editing. ML: Conceptualization, Data curation, Funding acquisition, Investigation, Project administration, Resources, Supervision, Writing – review & editing.
