# Supplementary material for: Conventional management has a greater negative impact on Phaseolus vulgaris L. rhizobia diversity and abundance than water scarcity
Source: Front Plant Sci. 2024 Jul 1;15:1408125. doi: 10.3389/fpls.2024.1408125 (PMC11246888; doi:10.3389/fpls.2024.1408125)
Supplement: Supplementary file 1 [file Table_1.docx]

Supplementary Material

**Conventional management has a greater negative impact on *Phaseolus* *vulgaris* L. rhizobia diversity and abundance than water scarcity**

**Arantza del-Canto^1*^, Álvaro Sanz-Sáez^2^, Katy D. Heath^3^, Michael A. Grillo^3,4^, Jonathan Heras^5^, Maite Lacuesta^1*^**

^1^Department of Plant Biology and Ecology, Pharmacy Faculty, University of the Basque Country, UPV/EHU, Paseo de la Universidad nº 7, Vitoria-Gasteiz, Spain.

^2^Department of Crop, Soil, and Environmental Sciences, Auburn University, 201 Funchess Hall, Auburn, AL 36849, USA.

^3^Department of Plant Biology, University of Illinois, 505 S. Goodwin Ave, Urbana, IL 61801, USA.

^4^Department of Biology, Loyola University Chicago, 1032 W. Sheridan Rd, Chicago, IL, 60618, USA.

^5^ Department of Mathematics and Computer Science, University of La Rioja, Ed. CCT, C, Madre de Dios 53, E-26004 Logroño, Spain.

**Supplementary Table S.1:** Origin of the studied local common genotypes indicating the annual mean pluviometry (mm), and the accumulated pluviometry mean (mm) during the common bean growing season (AA: Arrocina de Álava; AK: Amarilla de Kuartango; CL: Canela de León; MU: Morada de Usansolo; NB: Negra de Basaburua; PA: Pinta alavesa; RL: Riñón de León; y VO: Verde de Orbiso).

|  | **Origin locality** | **Accumulated annual pluviometry mean (mm)** | **Accumulated pluviometry means from May to September (mm)** |
| --- | --- | --- | --- |
|  |  |  |  |
| CL^3^ | La Bañeza (León) | 331.7 | 110.6 |
| RL^3^ | La Bañeza (León) | 331.7 | 110.6 |
| AK^1^ | Kuartango (Álava) | 716.2 | 172.2 |
| AA^1^ | Llanada Alavesa | 738.6 | 179.5 |
| PA^1^ | Llanada Alavesa | 738.6 | 179.5 |
| VO^1^ | Orbiso (Álava) | 858.6 | 200.8 |
| MU^1^ | Usánsolo (Vizcaya) | 1407.4 | 269.9 |
| NB^2^ | Basaburua (Navarra) | 1292.7 | 308.1 |
| ^1^Data represents the average precipitation from 2016 to 2021 obtained from Euskalmet (Basque Meteorological Agency). | | | |
| ^2^Data represents the average precipitation from 1999 to 2019 obtained from Meteo Navarra.  3 Data represents the average precipitation from 2016 to 2021 obtained from ITACyL (Instituto Tecnológico Agrario de Castilla y León). | | | |

**Supplementary Table S.2:** Number of isolated bacteria, average stability of clusters, mean values (±SE) and ANOVA results (p-value) of strain diversity indices (Shanon, Margalef and Pielou) and number of obtained clusters at a similarity level of 70% according to agricultural management (conventional and organic) and water availability (I, irrigated; and R, rainfed) (* p <0.05; ** p <0.01 and *** p <0.001; NS: non-significant).

|  |  |  | **Isolated bacteria** | **Cluster stability (bootstraping)** | **Cluster structure** | | **Low final level of similarity (%)** | **Diversity (Shannon´s index)** | **Richness (Margalef´s index)** | **Evenness (Pielou´s index)** | **Number of clusters** |
| --- | --- | --- | --- | --- | --- | --- | --- | --- | --- | --- | --- |
|  |  |  |  |  |  |  |  |  |  |  |  |
| **Conventional** | | | 127 | 0.629 | 90 of 1 strain | | 5 | 4.587±0.10 b | 105.794±1.11 b | 0.983±0.003 a | 106±1.41 b |
| **Organic** | | | 193 | 0.628 | 172 of 1 strain | | 6 | 5.147±0.04 a | 179.81±0.83 a | 0.991±0.002 a | 180±0.83 a |
| **Irrigation** | | | 167 | 0.633 | 145 of 1 strain | | 6 | 4.996±0.08 a | 153.805±1.17 a | 0.991±0.003 a | 154±1.26 a |
| **Rainfed** | | | 153 | 0.629 | 126 of 1 strain | | 2 | 4.8±0.14 a | 135.801±1.82 a | 0.987±0.003 a | 136±1.80 a |
| **Conventional** | | **I** | 71 | 0.639 | 61 of 1 strain | | 2 | 4.131±0.10 a | 64.765±1.42 a | 0.989±0.006 a | 65±2.09 a |
|  |  | **R** | 56 | 0.629 | 36 of 1 strain | | 2 | 3.768±0.15 a | 45.752±1.54 a | 0.984±0.006 a | 46±1.63 a |
| **Organic** | | **I** | 96 | 0.633 | 89 of 1 strain | | 8 | 4.501±0.05 a | 91.781±0.88 a | 0.995±0.004 a | 92±0.87 a |
|  |  | **R** | 97 | 0.636 | 91of 1 strain | | 1 | 4.503±0.08 a | 92.781±1.51 a | 0.993±0.000 a | 93±1.50 a |
|  |  |  |  |  |  |  | **ANOVA RESULTS** | | | |  |
|  |  |  |  |  |  |  | Factors | P-value |  |  |  |
|  |  |  |  |  |  |  | M | *** | *** | NS | *** |
|  |  |  |  |  |  |  | WA | NS | NS | NS | NS |
|  |  |  |  |  |  |  | M*WA | NS | NS | NS | NS |

**Supplementary Table S.3:** Number of isolated bacteria, strain diversity indices (Shanon. Margalef and Pielou) and number, structure and average stability of clusters obtained at a similarity level of 70% according to agricultural management (C, conventional; O, organic), water availability (I, irrigated; R, rainfed), and genotype (AA, Arrocina de Álava; AK, Amarilla de Kuartango; N, Negrita; NB, Negrita de Basaburua; RL, Riñon de Leon).

|  |  |  | Isolated bacteria | Diversity (Shannon´s index) | Richness (Margalef´s index) | Evenness (Pielou´s index) | Number of clusters | Cluster structure | Cluster stability (bootstraping) |
| --- | --- | --- | --- | --- | --- | --- | --- | --- | --- |
|  |  |  |  |  |  |  |  |  |  |
| AA | C | I | 10 | 2.303 | 9.565 | 1 | 10 | All of 1 strain | 0.648 |
|  |  | R | 15 | 2.488 | 12.631 | 0.97 | 13 | 12 of 1 strain | 0.641 |
|  | O | I | 21 | 3.045 | 20.671 | 1 | 21 | All of 1 strain | 0.634 |
|  |  | R | 19 | 2.944 | 18.660 | 1 | 19 | All of 1 strain | 0.648 |
|  |  | **Mean** | **65** | **4.102** | **61.760** | **0.993** | **60** | **60 of 1 strain** | **0.633** |
| AK | C | I | 10 | 2.303 | 9.566 | 1 | 10 | All of 1 strain | 0.637 |
|  |  | R | 6 | 1.792 | 5.442 | 1 | 6 | All of 1 strain | 0.671 |
|  | O | I | 20 | 2.831 | 17.666 | 0.979 | 18 | 9 of 1 strain | 0.647 |
|  |  | R | 18 | 2.890 | 17.654 | 1 | 18 | All of 1 strain | 0.6483 |
|  |  | **Mean** | **54** | **3.928** | **51.749** | **0.994** | **52** | **51 of 1 strain** | **0.634** |
| N | C | I | 18 | 2.700 | 15.650 | 0.976 | 16 | 15 of 1 strain | 0.65 |
|  |  | R | 11 | 2.398 | 10.583 | 1 | 11 | All of 1 strain | 0.65 |
|  | O | I | 18 | 2.890 | 17.654 | 1 | 18 | All of 1 strain | 0.638 |
|  |  | R | 24 | 3.178 | 23.685 | 1 | 24 | All of 1 strain | 0.64 |
|  |  | **Mean** | **71** | **4.177** | **66.765** | **0.993** | **66** | **64 of 1 strain** | **0.634** |
| NB | C | I | 16 | 2.773 | 15.639 | 1 | 16 | All of 1 strain | 0.631 |
|  |  | R | 9 | 2.043 | 7.545 | 0.982 | 8 | 7 of 1 strain | 0.676 |
|  | O | I | 16 | 2.773 | 15.639 | 1 | 16 | All of 1 strain | 0.646 |
|  |  | R | 21 | 3.045 | 20.672 | 1 | 21 | All of 1 strain | 0.642 |
|  |  | **Mean** | **62** | **4.082** | **59.758** | **0.996** | **60** | **58 of 1 strain** | **0.639** |
| RL | C | I | 17 | 2.639 | 14.647 | 0.974 | 21 | All of 1 strain | 0.642 |
|  |  | R | 15 | 2.610 | 13.631 | 0.991 | 15 | 14 of 1 strain | 0.653 |
|  | O | I | 21 | 2.979 | 19.672 | 0.994 | 20 | 19 of 1 strain | 0.64 |
|  |  | R | 15 | 2.700 | 14.631 | 1 | 15 | All of 1 strain | 0.66 |
|  |  | **Mean** | 68 | 4.061 | 60.763 | 0.988 | 61 | 56 of 1 strain | 0.636 |
|  |  |  |  |  |  |  |  |  |  |

**
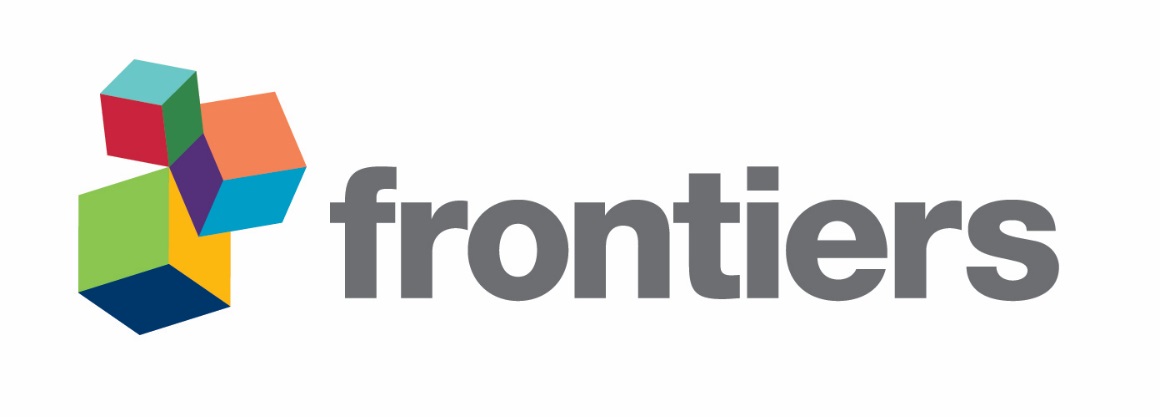
**
